# Predicting risk of ascending thoracic aortic aneurysm in asymptomatic adults using machine learning

**DOI:** 10.1371/journal.pone.0342482

**Published:** 2026-02-12

**Authors:** Seung Jae Lee, Jin Hee Ahn, Eun-Ah Cho, Suyong Jeon, Eun Jung Oh, Jae-Geum Shim

**Affiliations:** 1 Division of Cardiology, Department of Internal Medicine, Kangbuk Samsung Hospital, Sungkyunkwan University School of Medicine, Seoul, Republic of Korea; 2 Department of Anesthesiology and Pain Medicine, Kangbuk Samsung Hospital, Sungkyunkwan University School of Medicine, Seoul, Republic of Korea; 3 Healthcare Data Center, Kangbuk Samsung Hospital, Sungkyunkwan University School of Medicine, Seoul, Republic of Korea; Universitaria di Bologna, ITALY

## Abstract

Most patients with ascending thoracic aortic aneurysms (ATAA) remain asymptomatic until they develop fatal complications, including aortic dissection and rupture. We aimed to develop and validate machine-learning models for predicting ATAA risk. We developed a predictive model for the risk of ATAA based on data from 18,382 participants from the Kangbuk Samsung Health Study between January 1, 2010, and December 31, 2018. In the screening context, an ATAA was defined as an ascending thoracic aorta with a diameter ≥ 3.7 cm. For the model inputs, we used 16 variables from medical records, including basic patient information, physical indices, baseline medical conditions, and laboratory data at an early stage. A feature importance analysis was performed to analyze the factors related to the risk of ATAA in healthy adults. A machine learning model for predicting the risk of ATAA was developed using a 5-layer deep neural network (DNN) with the 15 key features. The performance of this model was evaluated in terms of accuracy, sensitivity, specificity, and area under the receiver operating characteristic curve (AUROC). Age was the most important factor in predicting the risk of ATAA, followed by hypertension, waist circumference, creatinine level, smoking, and body mass index. The AUROC and accuracy of our 5-layer DNN with the 15 key features are 80.4% and 83.5%, respectively. The sensitivity and specificity of the DNN were 69.4% and 81.1%, respectively. We developed and validated a machine learning model that can be used to assess the risk of ATAA. This model has potential applications in disease screening for ATAA at an early stage.

## Introduction

An early diagnosis of an ascending thoracic aortic aneurysm (ATAA) is challenging because it is asymptomatic enough to be termed a “silent killer.” [1]{Saeyeldin, 2019 #301}{Saeyeldin, 2019 #301}{Saeyeldin, 2019 #301}{Saeyeldin, 2019 #301}{Saeyeldin, 2019 #301} Previous studies show that ATAA progresses slowly: approximately 1 mm of growth in diameter is reported per year [2]. Except for cases with risk factors like bicuspid aortic valve, Marfan syndrome, or a family history of aortic dissections, it is often incidentally found during routine screening [3]. An ATAA can cause life-threatening complications such as aortic dissection or rupture, but early detection allows for medical treatment opportunities or preventive surgical replacement. Currently, echocardiography and computed tomography (CT) are the main methods used to measure aortic diameter. Transthoracic echocardiography can only measure the root of the aorta, while transesophageal echocardiography can detect the ascending and descending aorta with the limitation of observing the aortic arch [4]. Therefore, CT is the most widely used technique for visualizing the ascending thoracic aorta and diagnosing an ATAA. Due to the short scan times, which reduce respiratory and cardiac motion artifacts, CT scans can be helpful for getting diagnostic images of the entire thoracic aorta [5] However, CT scans to confirm abnormalities in the entire thoracic aorta are not suitable for population.-based screening tests because of the shortcomings of radiation exposure. Currently, there is no suitable tool for the routine screening of ATAA [6].

Machine learning has become essential in most sciences because of advancements in solving complex problems. Machine learning plays an important role in disease prediction and decision-making in medicine [7]. Recently, large amounts of medical data have been stored and utilized in real time, allowing machine learning techniques to outperform traditional statistical methods in handling data with strong complexity and nonlinearity. The application of machine learning can help screen individuals in high-risk groups for specific diseases and can be implemented in large populations. For instance, Yu et al. used the support vector machine classifier to estimate the diameter of the descending thoracic aorta with an error less than 2 mm^2^ [8]. Similarly, Mori et al. presented a predictive model based on logistic regression for an ATAA and demonstrated an AUROC value of 0.72 using only demographic and combination information [9]. Also, a deep learning model was used for semantic segmentation of the ascending and descending thoracic aorta in cardiac magnetic resonance images, specifically the U-Net architecture, to estimate the dimensions of the ascending and descending thoracic aorta [10].

To present a predictive model as a screening tool in the preliminary stage to proceed with CT for the final diagnosis of ATAA, we aimed to develop machine learning models and validate them internally. In addition, machine learning-based feature importance analysis was conducted on basic demographic and clinical information measured in healthy adults to identify the risk factors for ATAA.

## Methods

### Data sets

This study was approved by the Institutional Review Board of Kangbuk Samsung Hospital (IRB 2023-05-034), which waived the requirement for informed consent due to the retrospective design of the study. This study, as part of the Kangbuk Samsung Health Study, included participants who underwent comprehensive health screening examinations at the Kangbuk Samsung Hospital Total Healthcare Centers in Seoul, South Korea, between January 1, 2010, and December 31, 2018. The data for this study were accessed on March 5, 2023.

We collected basic patient information, physical indices, initial examination results, comorbid diseases, and laboratory tests at the time of the comprehensive health screening examinations. All ATAA and non-ATAA patients were identified using chest CTs. Sixteen predictor variables were obtained from both ATAA and non-ATAA patients and used to develop a predictive model. Basic patient information included age, sex, and smoking status. The initial examination findings included the mean values of systolic and diastolic blood pressure and heart rate when visiting the hospital. The comorbid diseases included diabetes mellitus, dyslipidemia, and hypertension. Laboratory tests included creatinine, glucose, high-sensitivity C-reactive protein (hs-CRP), and low-density lipoprotein (LDL).

In this study, an ATAA was defined as an ascending thoracic aorta of a diameter ≥ 3.7 cm in the screening context. Given that previous studies have shown that the mean aorta diameter in Koreans was less than 3.5 cm in both men and women, a threshold of 3.7 cm was chosen to maximize the sensitivity of detecting an enlarged aorta [11]. If multiple results of the chest CT scan in the same patient existed, only the test results conducted on the earliest date were included in our study.

### Data preprocessing

In the datasets, missing values were noted in < 2% of the records. To impute missing data, we replaced the missing values of each variable with the mean of the available values of that variable. The total dataset was split into training, validation, and test sets in a stratified manner on an ATAA basis to prevent contamination of the validation and test sets with the training data. We used 60% of the participants for training and 20% for validation and testing. Subsequently, we standardized the training set to maintain the parameters (mean and standard deviation values) for each feature, and these parameters were applied to transform the test set. In the binary classification problem with the risk of an ATAA in this study, most patients would have no disease, and detecting the disease is of greater interest. To address the large class imbalance problem, a synthetic minority oversampling technique was applied [12].

### Feature importance analysis and feature selection

We performed feature importance analysis using random forest, eXtreme Gradient Boosting (XGBoost), and AdaBoost algorithms to select those features that contribute most to our prediction variable (ATAA) [13]. After calculating the feature importance by applying each machine learning algorithm, the overall feature importance was determined by averaging them. We chose the optimal number of features as the input in our machine learning model while changing the number of features according to the overall feature importance.

### Machine learning model building

We developed a deep neural network (DNN) model to predict ATAA in patients. In our predictive model, the DNN consists of five layers: an input layer, an output layer, and three hidden layers. The input layer obtains data from the features ranked according to their average feature importance. We estimated the number of features required to train the DNN to be 16. We used TensorFlow to build a DNN with the Adam optimizer and a binary cross-entropy cost function with a learning rate of 0.001 and a batch size of 128 [14].{Saeyeldin, 2019 #301}{Abadi, 2016 #188}{Abadi, 2016 #188}{Abadi, 2016 #188}{Abadi, 2016 #188}{Abadi, 2016 #188}{Chang, 2020 #185}{Abadi, 2016 #188}{Abadi, 2016 #188}.

Python 3.7.13 (Python Software Foundation, Wilmington, DE, USA) and R version 4.0.3 (R Foundation for Statistical Computing, Vienna, Austria) were used for the DNN model development and descriptive statistics.

### Performance evaluation

We tested the prediction performance of our proposed 5-layer DNN model with a hold-out dataset (n = 3,677) that was not used for training or validation to provide an unbiased assessment. To compare the prediction performance of the DNN model with that of other external machine learning models, we separately trained the following models: decision tree, random forest, XGBoost, and AdaBoost. The prediction performance of the DNN model was compared with that of other machine-learning models, including decision tree, random forest, XGBoost, and AdaBoost. To determine the goodness of fit of the medical predictive model, we used the area under the receiver operating characteristic curve (AUROC), accuracy, sensitivity, and specificity.

## Results

A total of 18,382 patients who visited Kangbuk Samsung Hospital Total Healthcare Centers in Seoul, Korea, were analyzed in our study. 1,147 patients (6.24%) experienced an ATAA according to our definition. Table 1 shows the characteristics of the subjects with and without an ATAA. The mean ages of ATAA and non-ATAA participants were 49.9 ± 9.5 and 40.5 ± 6.7 years, respectively. Although the percentage of males with an ATAA was higher (93.7%) than those without(90.6%), the difference in sex between patients with and without ATAA was not large.

**Table 1:** Patient demographic data and variable features.

Variables	Non-ATAA (n = 17,235)	ATAA (n = 1,147)	p-value
Basic patient information			
Age, years	40.5 ± 6.7	49.9 ± 9.5	<.001
Gender, female, n (%)	15,622 (90.6)	1,075 (93.7)	0.001
Smoking history, n (%)	13,896 (80.6%)	908 (79.2%)	0.24
Physical index			
BMI, kg/m^2^	24.0 ± 3.0	25.5 ± 3.0	<.001
BSA, m^2^	1.8 ± 0.2	1.9 ± 0.2	<.001
Waist circumference, cm	83.8 ± 7.8	88.3 ± 7.6	<.001
Initial examination findings			
Systolic blood pressure, mmHg	116.0 ± 11.6	121.9 ± 13.2	<.001
Diastolic blood pressure, mmHg	73.9 ± 8.6	78.9 ± 9.6	<.001
Heart rate, beats per minute	64.5 ± 9.0	64.6 ± 9.7	0.71
Comorbid diseases, n (%)			
Diabetes Mellitus	455 (2.6)	81 (7.1)	<.001
Dyslipidemia	1,342 (7.8)	148 (12.9)	<.001
Hypertension	1,356 (7.9)	325 (28.3)	<.001
Laboratory tests			
Creatinine, mg/dL	0.9 ± 0.1	1.0 ± 0.2	0.004
Glucose, mg/dL	94.9 ± 14.5	99.0 ± 20.0	<.001
hs-CRP, mg/dL	0.1 ± 0.3	0.2 ± 0.5	0.03
LDL, mg/dL	121.7 ± 30.3	125.0 ± 30.6	<.001

ATAA = ascending thoracic aortic aneurysm, BMI = body mass index, BSA = body surface area, hs-CRP = high-sensitivity C-reactive protein, LDL = low-density lipoprotein.

### Feature selection

**Fig 1:**
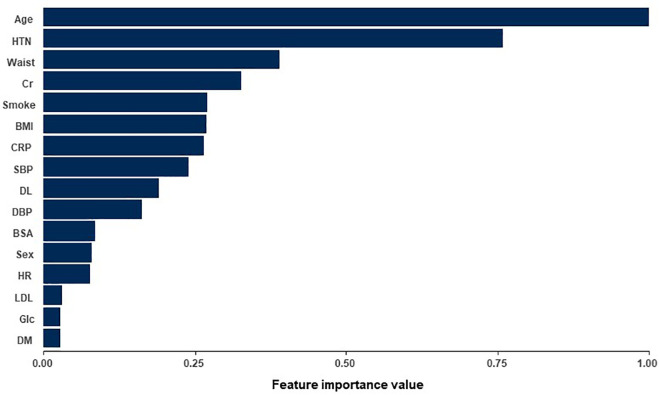
Results of normalized feature importance analysis. HTN, hypertension; Cr, creatinine; BMI, body mass index; CRP, C-reactive protein; SBP, systolic blood pressure; DL, dyslipidemia; DBP, diastolic blood pressure; BSA, body surface area; HR, heart rate; LDL, low-density lipoprotein; Glc, glucose; DM, diabetes mellitus.

**Fig 2:**
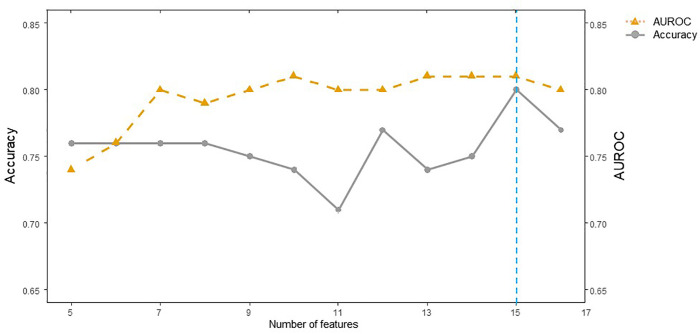
The influence of the number of features on validation accuracy and AUROC. AUROC, area under the receiver operating characteristic curve.

Fig 1 shows the average feature importance values obtained from the random forest, XGBoost, and AdaBoost algorithms. Age was the most important factor in predicting the risk of ATAA, followed by hypertension, waist circumference, creatinine level, smoking, and body mass index. Additionally, we evaluated the predictive performance of the validation dataset in terms of accuracy and AUROC. We found that the use of the top 15 features for DNN model accuracy led to AUROCs of 0.804 and 0.835, as shown in Fig 2.

### Performance of the AI prediction model

**Fig 3:**
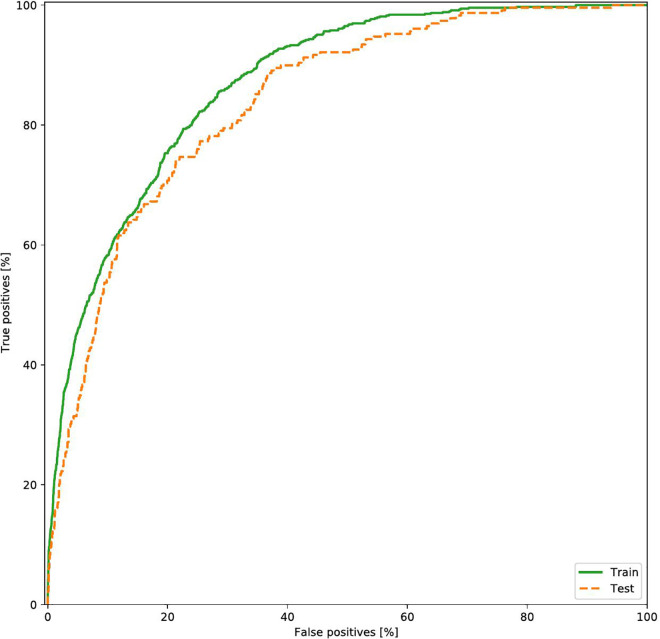
The prediction performance of the DNN model (testing data set).

The prediction performance of the DNN model with the isolated testing dataset (n = 3,677) is presented in Table 2 and Fig 3. Our proposed model, with the 15 key features, showed a sensitivity of 0.694, specificity of 0.811, accuracy of 0.804, and AUROC of 0.835. When we compared the performance metrics of our model with those of other machine learning algorithms, we observed that our proposed 5-layer DNN model was superior to the other models in terms of sensitivity and AUROC.

**Table 2:** Summary of five machine learning models with testing data.

Model	TN	FP	FN	TP	SN	SP	AC	AUROC
5-layer DNN	2796	652	70	159	0.694	0.811	0.804	0.835
Decision Tree	3140	308	171	58	0.253	0.911	0.870	0.582
Random Forest	2487	961	73	156	0.681	0.721	0.719	0.775
XGBoost	3348	100	186	43	0.188	0.971	0.922	0.814
AdaBoost	2621	827	72	157	0.686	0.760	0.756	0.765

TN, true negative; FP, false positive; FN, false negative; TP, true positive; SN, sensitivity; SP, specificity; AC, accuracy; AUROC, area under the receiver operating characteristic curve.

## Discussion

The thoracic aorta is a large artery in the body that consists of three parts: the ascending aorta, aortic arch, and descending aorta. Enlargement of the aorta only mildly represents ectasia, and an aneurysm may occur because of localized weakness of the arterial wall when ectasia exceeds tolerance limits [15,16]. Thoracic aortic aneurysms can occur in one or more parts of the aorta. According to a previous study, approximately 60% of thoracic aortic aneurysms occur in the aortic root and ascending aorta, and the remaining 40% are related to the descending aorta [17]. Considering that the proportion of ATAA in thoracic aortic aneurysms is not small, it is essential to reduce the underdiagnosis of ATAA by using highly sensitive tests, even if the specificity is slightly compromised.

As a test for routine screening of population size, we should focus on increasing the sensitivity of the test, considering fatal complications. Currently, computed tomography angiography (CTA) or magnetic resonance angiography (MRA) are the selected imaging tests for diagnosing ATAA and measuring its severity [18]. However, CTA involves radiation exposure and the use of contrast dye, with the limitations of reduced renal function. As MRA is time-consuming and involves the use of a contrast dye, there are limitations for patients with claustrophobia or kidney problems. Therefore, we believe that a screening tool that can be easily applied will help reduce underdiagnosis by first selecting high-risk patients and then using additional imaging methods.

Considering the indolent and asymptomatic characteristics of ATAA, emergency surgery is always possible in the event of complications. However, the mortality rate is estimated at 20% in the case of emergency surgery, whereas there was a low risk of mobility and morbidity in the case of elective surgery [19]. Therefore, it is imperative to actively try to detect an ATAA in advance so that no acute event occurs. Presently, we don’t exactly understand the benefit of surgical intervention compared with surveillance based on the size of the dilated ascending aorta [20]. However, this means that early identification of patients at high risk of mortality is important for the treatment of ATAA patients.

**Fig 4:**
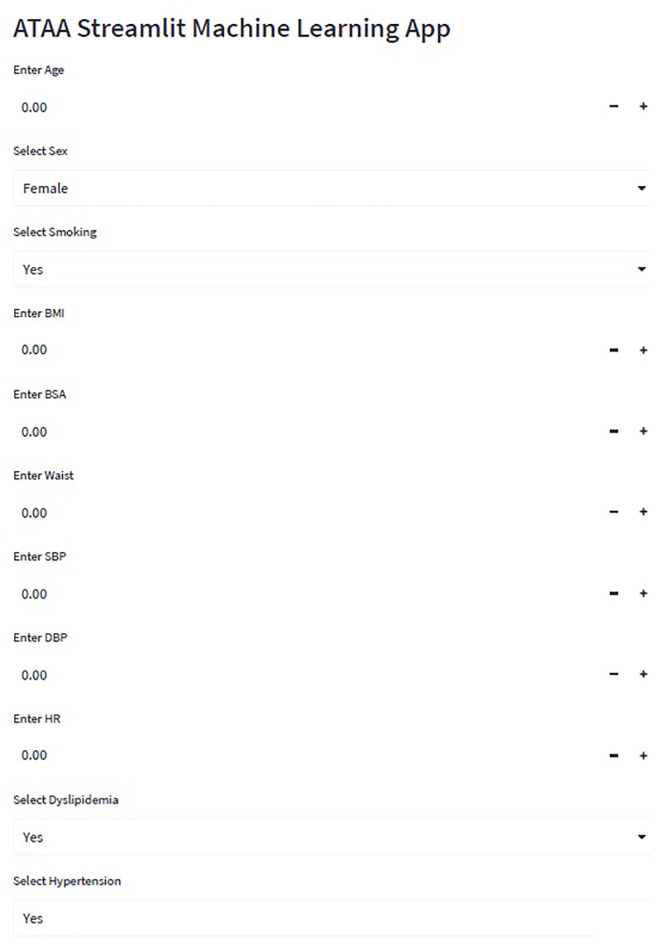
Screenshot of the web application.

In this study, we propose an ATAA prediction model that uses a deep learning algorithm for healthy adults. Fifteen input values were required to apply the proposed prediction model. We also developed a web application that allows anyone to use our model to assess the risk of ATAA (Fig 4). Basic patient information, vital signs, comorbidities, and laboratory test results are typically obtained when visiting a hospital for the first time. We anticipate that our ATAA risk assessment approach will assist in directing future testing to only those patients who need intensive care.

## Limitations

Our study has some limitations. To test the performance of our model, we validated it using an isolated test dataset split from the entire dataset before the training stage. However, to generalize our proposed model, external validation using new data from other hospitals or countries are required. We expect that it will be helpful to check the results of external data using our web application. In addition, the subjects of this study were only healthy adults who visited the hospital for medical checkups; therefore, there is a possibility of selection bias. Therefore, a performance improvement can be expected when training the model using a wide range of data, including people for health screening and other patients with illnesses. Furthermore, although family history of aortic disease is a well-established risk factor for ATAA, we could not incorporate this variable into our machine learning model. Due to the retrospective nature of the study and the specific format of the health screening data, detailed information regarding family history of aortic disease was not available. We acknowledge that the lack of this genetic and familial background information may have limited the sensitivity of our model. Future prospective studies should aim to include detailed family history data to potentially improve the predictive performance and clinical applicability of the model. Finally, our study was designed using only retrospective data, but we should check the actual performance through a prospective study in the future to confirm its clinical usefulness.

## Conclusions

In conclusion, we developed and validated our machine learning model using 15 selected features based on patient demographics and clinical information. We also created a web application for anyone who wants to estimate the risk of an ATAA to access our model. This algorithm may be useful for screening unidentified ATAA.
